# Increased anxiety in corticotropin-releasing factor type 2 receptor-null mice requires recent acute stress exposure and is associated with dysregulated serotonergic activity in limbic brain areas

**DOI:** 10.1186/2045-5380-4-1

**Published:** 2014-01-21

**Authors:** Orna Issler, Roderick N Carter, Evan D Paul, Paul AT Kelly, Henry J Olverman, Adi Neufeld-Cohen, Yael Kuperman, Christopher A Lowry, Jonathan R Seckl, Alon Chen, Pauline M Jamieson

**Affiliations:** 1 Department of Neurobiology, Weizmann Institute of Science, Rehovot 76100, Israel; 2 Centre for Cardiovascular Science, Queens Medical Research Institute, 47 Little France Crescent, Edinburgh EH16 4TJ, UK; 3 Department of Integrative Physiology and Center for Neuroscience, University of Colorado Boulder, Boulder, CO 80309, USA; 4 Centre for Cognitive and Neural Systems, University of Edinburgh, 1 George Square, Edinburgh EH8 9JZ, UK

**Keywords:** 5-HT_1A_ receptor, Anxiety, Corticotropin-releasing factor type 2 receptor, Raphe nuclei, Serotonin, Stress

## Abstract

**Background:**

Corticotropin-releasing factor type 2 receptors (CRFR2) are suggested to facilitate successful recovery from stress to maintain mental health. They are abundant in the midbrain raphe nuclei, where they regulate serotonergic neuronal activity and have been demonstrated to mediate behavioural consequences of stress. Here, we describe behavioural and serotonergic responses consistent with maladaptive recovery from stressful challenge in CRFR2-null mice.

**Results:**

CRFR2-null mice showed similar anxiety levels to control mice before and immediately after acute restraint stress, and also after cessation of chronic stress. However, they showed increased anxiety by 24 hours after restraint, whether or not they had been chronically stressed.

Serotonin (5-HT) and 5-hydroxyindoleacetic acid (5-HIAA) contents were quantified and the level of 5-HIAA in the caudal dorsal raphe nucleus (DRN) was increased under basal conditions in CRFR2-null mice, indicating increased 5-HT turnover. Twenty-four hours following restraint, 5-HIAA was decreased only in CRFR2-null mice, suggesting that they had not fully recovered from the challenge. In efferent limbic structures, CRFR2-null mice showed lower levels of basal 5-HT in the lateral septum and subiculum, and again showed a differential response to restraint stress from controls.

Local cerebral glucose utilization (LCMRglu) revealed decreased neuronal activity in the DRN of CRFR2-null mice under basal conditions. Following 5-HT receptor agonist challenge, LCMRglu responses indicated that 5-HT_1A_ receptor responses in the DRN were attenuated in CRFR2-null mice. However, postsynaptic 5-HT receptor responses in forebrain regions were intact.

**Conclusions:**

These results suggest that CRFR2 are required for proper functionality of 5-HT_1A_ receptors in the raphe nuclei, and are key to successful recovery from stress. This disrupted serotonergic function in CRFR2-null mice likely contributes to their stress-sensitive phenotype. The 5-HT content in lateral septum and subiculum was notably altered. These areas are important for anxiety, and are also implicated in reward and the pathophysiology of addiction. The role of CRFR2 in stress-related psychopathologies deserves further consideration.

## Background

Serotonin (5-HT) is a key neurotransmitter in the control of mood. It is the major target of current antidepressant medications, and often also of treatments for anxiety disorders [1,2]. The principal sources of 5-HT neurons projecting to the forebrain are the midbrain dorsal (DRN) and median (MRN) raphe nuclei [3,4].

Corticotropin-releasing factor (CRF) is a key mediator of the stress response [5-7], and anxiety and affective disorders have been associated with CRF hyperactivity [8]. Corticotropin-releasing factor receptors are abundant in both DRN and MRN [9-11], where they are expressed in serotonergic and non-serotonergic neurons, including regulatory GABAergic neurons [12,13], suggesting the potential for complex interactions between CRF and serotonergic systems. Electrophysiological studies show that exogenous CRF administered to the raphe modulates serotonergic neuronal firing activity [14-16], and therefore CRF receptor-mediated effects on stress-related behaviours may be mediated via 5-HT *in vivo*[17-20].

Type 1 (CRFR1) and type 2 (CRFR2) CRF receptors [21-23] are preferentially activated by CRF or urocortin neuropeptides (Ucn1, Ucn2, Ucn3), respectively [24-28]. The raphe nuclei receive inputs from both CRF and Ucn1 expressing neurons [14,15,29-31], and a potentially important role for the CRF system in controlling 5-HT neurons here is emerging.

CRFR2 is expressed at high levels in the raphe nuclei, while CRFR1 is expressed at lower levels in the raphe nuclei in rats and appears to be absent from this area in mice and human beings [9-11]. Exogenously administered CRFR2 agonists induce c-Fos expression in DRN 5-HT neurons, increase their firing rate, and increase 5-HT release in efferent stress-related nuclei [32-36]. In pharmacological studies, CRFR2 activation in the DRN potentiates immediate fear responses [35], fear conditioning and escape deficits 24 hours later in a model of learned helplessness [37,38], and decreases exploratory behaviours [19] in rodents. Recently, altered anxiety-like behaviour in Ucn-knockout or Ucn-overexpressing mice has been linked to disturbances in serotonergic activity in the neural circuitry controlling anxiety [39-41]. The Ucn1/Ucn2/Ucn3 triple knockout mouse phenotype suggests that CRFR2 and particularly Ucn3 are involved in successful recovery from stress [41]. This interaction with the 5-HT system may provide a major link between the two main arms of the central stress response; the CRF/Ucns peptidergic pathways and the sympathetic monoaminergic system.

5-HT_1A_ receptors (5-HT_1A_R) are also particularly associated with modulating anxiety [42] and pharmacological stimulation of CRF receptors in the raphe nuclei has been demonstrated to regulate serotonergic neuronal firing here [43,44]. Thus, CRF-containing neuronal projections from the central amygdala (CeA) to the raphe nuclei [45] may modulate activity at postsynaptic 5-HT_1A_R by directly regulating activity of efferent 5-HT projections or may have wider-ranging effects on 5-HT function via altered raphe 5-HT_1A_R autoreceptor activity. Conversely, 5-HT_1A_R activity can influence CRF-induced changes in behaviour; 5-HT_1A_R-selective agonists can attenuate CRF-induced grooming [46]. We have previously shown that 5-HT_1A_R responsiveness plays a key role in stress-related behaviours associated with chronic activation of CRFR2 [39] and that interaction is further explored in the studies presented here.

Activation of CRFR2 affects anxiety-like behaviour under stressed conditions [47-49] and CRFR2-null mice have an anxiogenic phenotype [50,51]. This raises the question of what role CRFR2 might play in the pathophysiology of anxiety-related and affective disorders in human beings. To further investigate the mechanisms underlying this, we examined the anxiety phenotype of CRFR2-null mice in detail, and characterized their serotonergic responses to stress.

## Methods

### Animals

Mice were housed in temperature- and lighting-controlled rooms (lights on, 12 h) with free access to laboratory chow and water. CRFR2-null mice, as previously described [50], and control littermates (C57BL6 × 129) were the adult male offspring of parents heterozygous for the knockout allele. For CRFR2 mRNA studies, adult male wild type C57BL6/J mice (Harlan Laboratories) were used. Mice were group housed, except for the chronic variable mild stress (CVMS) protocols, for which they were singly housed. Principles of laboratory animal care (NIH No. 85-23, 1985) were followed. All procedures were approved by The Weizmann Institute Animal Use and Care Committee or the United Kingdom Animals (Scientific Procedures) Act, 1986.

### Behavioural testing

**Figure 1:**
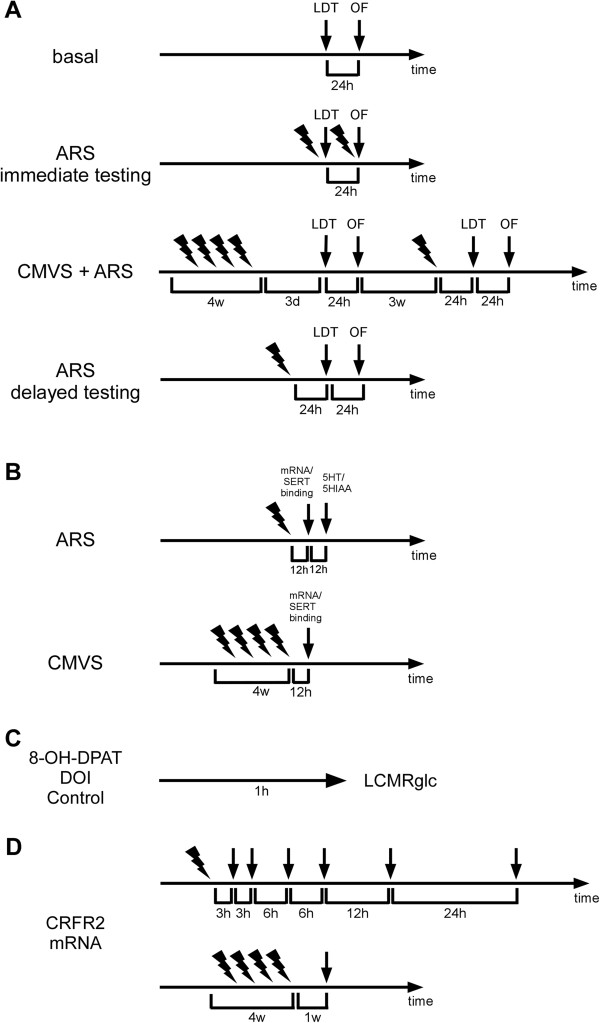
**Schematic representation of experimental protocols and timelines. (A)** Separate cohorts of CRFR2-null and control mice were tested for anxiety-like behaviour in the light/dark transfer and open-field tests: under basal conditions; immediately following ARS; following CVMS and again 24 to 48 h after an ARS applied 3 weeks after the end of CVMS; 24 to 48 h following ARS. **(B)** CRFR2-null and control mice were exposed to no stress, ARS or CVMS, and mRNA expression of stress-related genes and serotonin transporter (SERT) binding were quantified 12 h after the end of stress. 5-HT/5HIAA content in brain nuclei were quantified in unstressed mice and 24 h after ARS. **(C)** LCMRglu was measured in CRFR2-null and control mice one hour after administration of saline or 5-HTR agonist. **(D)** CRFR2 mRNA levels in brain were quantified over a 48-h time course following ARS or 7 days after CVMS in control mice. , ARS; , CVMS; d, days; w, weeks.

Tests were carried out during the dark phase of the light cycle on adult male mice (2 to 4 months). Mice were habituated in the home cage in a dark room for 2 hours prior to each behavioural test. Separate groups of mice were tested under: (a) basal conditions with no stress applied prior to testing, *n* = 12 for control group, *n* = 14 for CRFR2-null group; (b) immediately following 30 min of acute restraint stress (ARS), *n* = 13, both groups; (c) 24 to 48 hours following ARS, *n* = 5 for control group, *n* = 8 for CRFR2-null group (light/dark transfer test performed at 24 hours post-stress, open-field at 48 hours post-stress); (d) 3 to 4 days following a 4-week CVMS protocol, (light/dark transfer test performed at 3 days post-stress, open-field at 4 days post-stress), *n* = 10 for control group, *n* = 11 for CRFR2-null group. The mice of group d were then retested 3 weeks later, when an ARS was applied and testing was performed at 24 to 48 hours. Figure 1 shows the timeline of the experimental protocols with stress procedures.

### Open-field (OF) test

The apparatus and experimental conditions were as previously described [50]. Mice were placed in the centre of the apparatus to initiate a 10-min test session. Visits to, and distance travelled and time spent in the inner zone of the arena were quantified using a video tracking system (VideoMot2; TSE Systems, Bad Hamburg, Germany).

### Light/dark transfer test (LDT)

Apparatus and experimental conditions were as previously described [50]. During a 5-min test session, visits to, and distance travelled and time spent in the light compartment were measured.

### Stress procedures

Mice were subjected to 30 min ARS in a ventilated 50 ml plastic centrifuge tube. The CVMS regime was modified from Ducottet *et al.*[52]. Mice were singly housed and a variety of mild stressors were applied on an unpredictable schedule, 2 to 3 stressors per day for 4 weeks; these included disruptions to the light-dark cycle, cage shift to one previously inhabited by another male, cage tilt, damp bedding, low-intensity stroboscopic illumination, white noise, restraint stress, short periods of food or water restriction, and housing with no bedding followed by water in the cage. Controls were housed under stress-free conditions.

In the CVMS paradigm, mice were behaviourally tested 48 hours following termination of the last stressor, which was standardized and was 24 hours of constant light for all mice (*n* = 10 or 11). For *in-situ* hybridization and 5-HT transporter (SERT) binding studies, mice (*n* = 6 for control basal group, *n* = 8 for CRFR2-null basal group, *n* = 7 for all stress groups) were killed 12 hours after ARS, or after the last variable stressor, by decapitation within 15 s of disturbing the home cage. The brains were removed, rapidly frozen on dry ice and stored at −80°C until analysis.

### Local cerebral glucose utilization (LCMRglu)

Local cerebral glucose utilization (LCMRglu) was determined as described previously [39,53]. Mice (*n* = 8, all groups) were injected (intraperitoneally) with either 10 mg kg^-1^ 8-hydroxy-*N*,*N*-dipropyl-2-aminotetralin (8-OH-DPAT), 25 mg kg^-1^ 1-(2,5-dimethoxy-4-iodophenyl)-2-aminopropane (DOI) or vehicle (0.1 ml 0.9% NaCl). At 10 min after 8-OH-DPAT, or 20 min after DOI, 5 μCi [^14^C]-2-deoxyglucose in 0.4 ml 0.9% NaCl was injected intraperitoneally. After 45 min, mice were decapitated and their brains analyzed by quantitative autoradiographic imaging, as described previously [54,55].

### Analysis of tissue concentrations of 5-HT and 5-HIAA

Mice (*n* = 7 for unstressed groups, *n* = 6 for ARS groups) were killed by decapitation under basal conditions or 24 hours following ARS. Brains were stored at −80°C until analysis. Areas selected for microdissection were identified by comparison with a standard mouse brain stereotaxic atlas [56]. To ensure accuracy, we used a stereomicroscope to visualize neuroanatomical landmarks for use as reference points in identifying specific nuclei and subdivisions of the DRN. Small diameter microdissection tools (310 to 410 μm diameter) were used to restrict dissections to the subregion of interest. High-pressure liquid chromatography analysis of 5-HT and 5-hydroxyindoleacetic acid (5-HIAA) was performed, as previously described [57].

### CRFR2 mRNA qPCR analysis

Quantitative PCR for CRFR2 mRNA expression was carried out as previously reported [40] in brain taken from naïve mice (controls), or 3, 6, 12, 24 or 48 hours after ARS, or, for CVMS mice, one week after the end of the stress protocol (*n* = 8 all groups).

### *In-situ* hybridization (ISH) histochemistry

Coronal brain sections (10 μm) were cut on a cryostat, thaw-mounted onto silanized glass slides, and stored at −80°C until use. *In-situ* hybridization procedures and probes were as previously described [58-60]. Plasmids (generous gifts from Professor M. Holmes and Dr V. Bombail) containing cDNA fragments for glucocorticoid receptor (GR), mineralocorticoid receptor (MR), 5-HT_1A_ R, 5-HT_2C_R and tryptophan hydroxylase 2 (TPH2) were used to generate ^35^S-UTP-labelled specific antisense probes to mRNAs. Following ISH, slides were dipped in Kodak Autoradiography Emulsion (Molecular Imaging Systems, New York, USA) and exposed at 4°C for between 24 h and 6 weeks, depending on the probe, developed and counterstained. The hybridization signal for each brain area was determined using computer-assisted grain counting software (Zeiss KS 300 3.0, Carl Zeiss Vision, GmbH). For each animal, silver grains were counted in a fixed circular area over 6 to 10 individual neurons per subregion. The background, counted over areas of white matter, was subtracted. Analysis was carried out blind to treatment group.

### 5-HT transporter (SERT) binding

Serotonin transporter (SERT) binding was determined on brain sections, cut as above, using (^3^H)-paroxetine (Perkin Elmer, UK) as previously described [61]. Slides were then exposed to (^3^H)-sensitive film (Amersham Hyperfilm MP, GE Healthcare, UK) at −80°C for 6 weeks. Analysis of autoradiographs was performed by measuring the signal over the area of interest with densitometry software (MCID Basic 7.0, Imaging Research, Inc.). The background was subtracted.

### Statistical analyses

Statistical analyses employed the two-tailed Student’s *t* test or two-way analysis of variance (ANOVA) with post-hoc analysis using Fisher’s protected least significant difference test as appropriate, with the exception of time course of CRFR2 expression, where one-way ANOVA with Dunnett’s post-hoc analysis was used. Data are presented as mean ± standard error of the mean (SEM). Differences were considered statistically significant at *P* < 0.05.

## Results

### CRFR2-null mice show increased anxiety 24 to 48 h after acute restraint stress (ARS)

**Figure 2:**
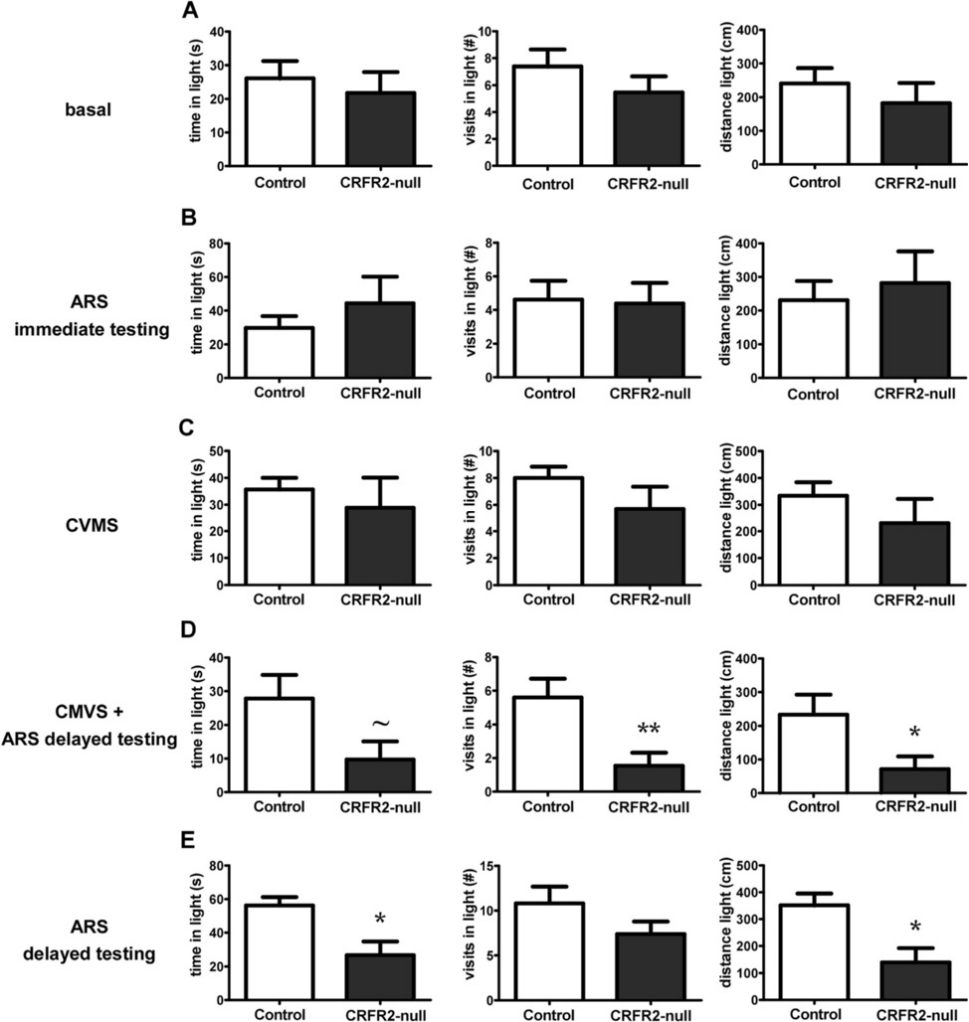
**CRFR2-null mice exhibit increased anxiety-like behaviour 24 hours after ARS in the LDT.** Under basal conditions **(A)**, immediately following ARS **(B)**, or after CVMS **(C)**, no differences were observed in behaviour between CRFR2-null mice and controls. However, when CVMS mice **(D)** or naïve mice **(E)** were exposed to ARS and tested 24 to 48 hours later, CRFR2-null mice showed increased anxiety-like behaviour compared with control mice. Data expressed as mean ± SEM. *N* = 10 to 14 for **A**-**D**, *N* = 5 to 8 for **E**. *P < 0.05, **P < 0.01, ^~^*P* = 0.53, compared with control.

**Figure 3:**
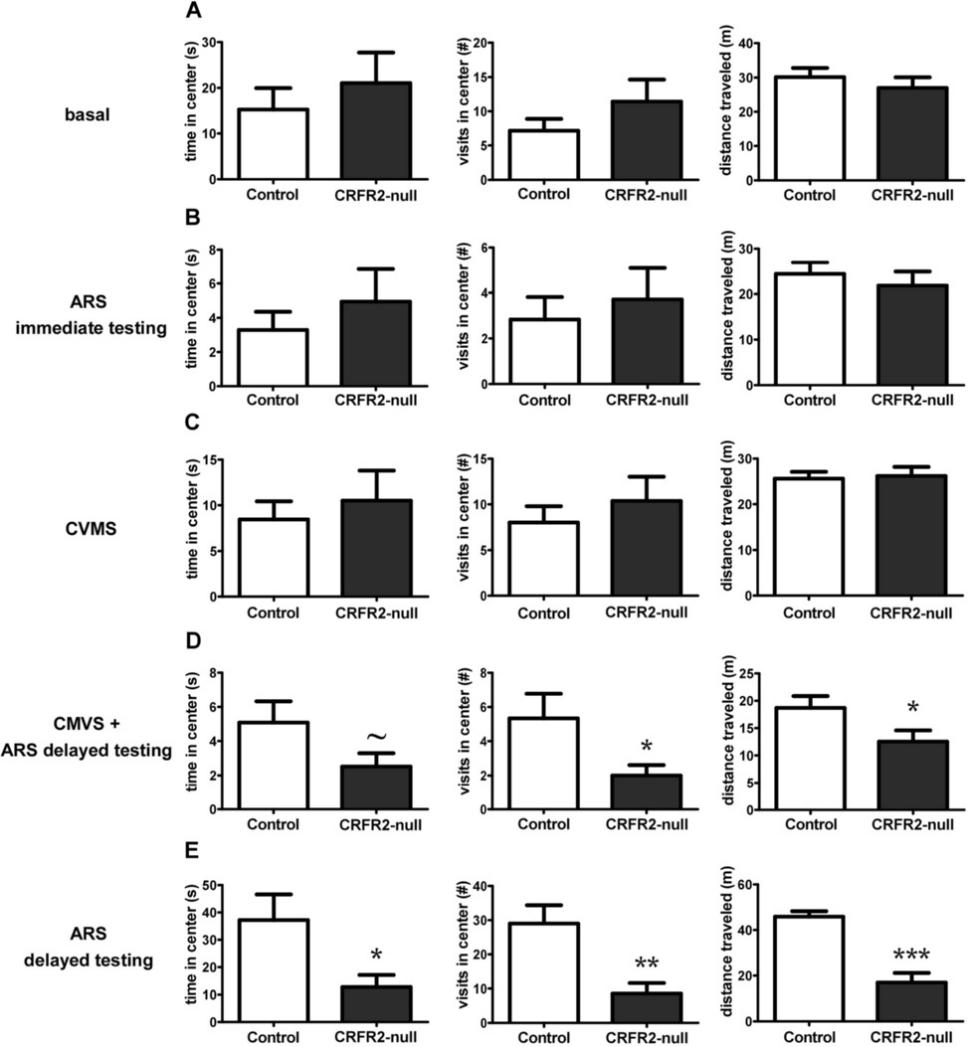
**CRFR2-null mice exhibit increased anxiety-like behaviour 48 hours after ARS in the OF test.** Under basal conditions **(A)**, immediately following ARS **(B)**, or after CVMS **(C)**, no differences were observed in behaviour between CRFR2-null mice and controls. However, when CVMS mice **(D)** or naïve mice **(E)** were exposed to ARS and tested 24 to 48 h later, CRFR2-null mice showed increased anxiety-like behaviour compared with control mice. Data expressed as mean ± SEM. *N* = 10 to 14 for **A**-**D**, *N* = 5 to 8 for **E**. *P < 0.05, **P < 0.01, ***P < 0.001, ^~^*P* = 0.085, compared with control.

Under basal conditions, where mice were not exposed to stress (other than that caused by the test itself), CRFR2-null mice and littermate controls showed no differences in anxiety-related behaviour in two well-validated behavioural tests, the LDT (Figure 2) and the OF test (Figure 3), compared with littermate controls.

Because this finding contrasted with previous reports [50,51], we hypothesized that stressful challenge was required to reveal the role of CRFR2 in anxiety. Another group of mice was tested immediately following 30 min ARS. Again, no effect of genotype on anxiety-like behaviour was observed (Figures 2 and 3). A further cohort of mice exposed to CVMS was tested 3 to 4 days after the end of the protocol, to allow for recovery from the final acute stressor, and again no differences were observed between control and CRFR2 mice in either behavioural test.

However, 3 weeks later, these same CVMS mice were exposed to a single 30-min ARS, and 24 to 48 h later the CRFR2-null mice showed significantly increased indices of anxiety compared with controls, with fewer visits to (*t* = 3.022, *P* = 0.007, *n* = 10 or 11), shorter distance travelled in (*t* = 2.360, *P* = 0.029, *n* = 10 or 11), and a trend to less time spent in the light chamber in the LDT (*t* = 2.062, *P* = 0.053, *n* = 10 or 11) (Figure 2), and fewer visits to the centre of (*t* = 2.271, *P* = 0.036, *n* = 10 or 11) and less time spent in (*t* = 2.231, *P* = 0.039, *n* = 10 or 11) the centre and a trend to less time spent in the OF test (*t* = 1.825, *P* = 0.085, *n* = 10 or 11) (Figure 3).

We then examined whether this delayed effect of ARS on anxiety was dependent on prior CVMS by subjecting a further cohort of mice to ARS alone, and observed the same increased anxiety-like behaviour 24 to 48 hours post-stress (Figures 2 and 3). In the LDT, CRFR2-null mice spent less time (*t* = 2.650, *P* = 0.023, *n* = 5 to 8) and travelled a shorter distance (*t* = 2.833, *P* = 0.016, *n* = 5 to 8) in the light chamber. In the OF test, CRFR2-null mice spent less time in (*t* = 2.675, *P* = 0.022, *n* = 5 to 8) and made fewer visits to the centre (*t* = 3.604, *P* = 0.004, *n* = 10 to 11), and travelled a shorter distance (*t* = 5.078, *P* = 0.0004, *n* = 10 to 11).

### Serotonergic function is altered in the raphe nuclei of CRFR2-null mice

**Figure 4:**
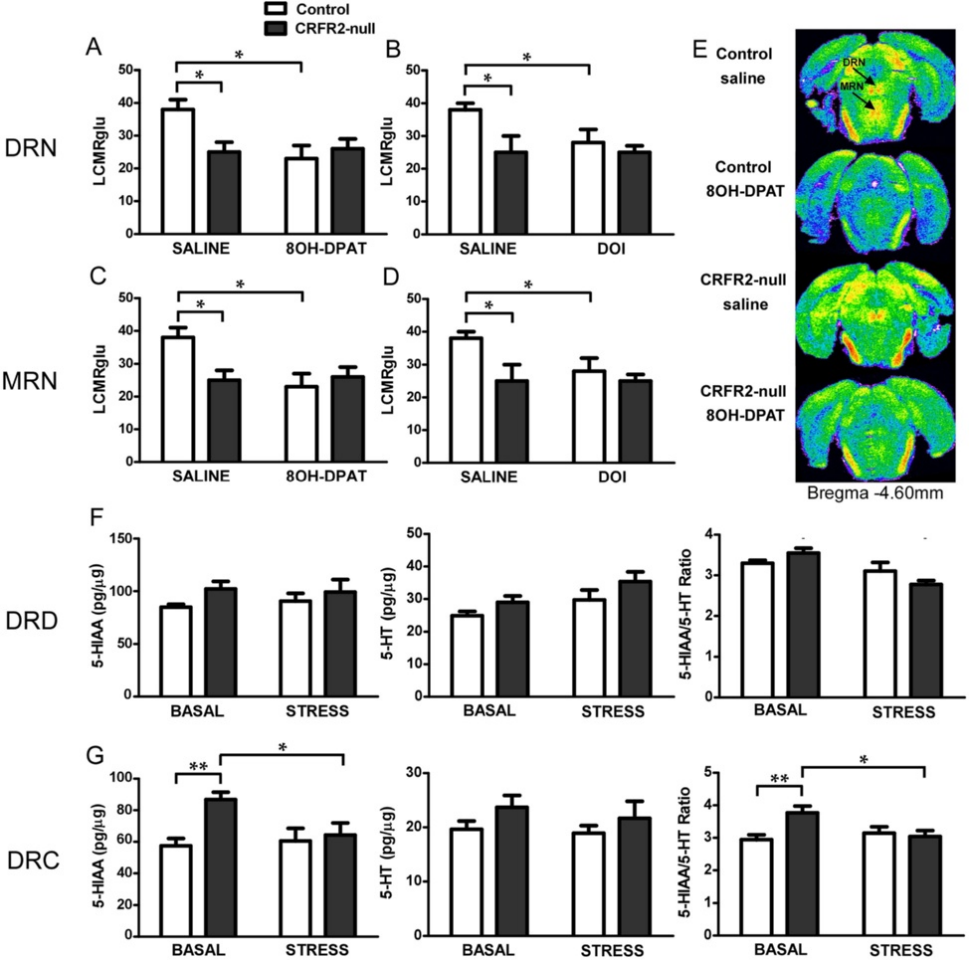
**Serotonergic function is altered in the raphe nuclei of CRFR2-null mice. (A-E)** LCMRglu in the dorsal raphe nucleus (DRN) and median raphe nucleus (MRN) is lower in CRFR2-null mice than controls under basal conditions. **(A,C)** 8-OH-DPAT or **(B,D)** DOI administration decreased LCMRglu in the **(A,B)** DRN or **(C,D)** MRN only in control mice. **(E)** Colour-coded autoradiograms from coronal brain sections at the level of the midbrain raphe. ‘Warm’ colours represent high levels of [^14^C]-2-deoxyglucose accumulation while ‘cold’ colours represent low tracer accumulation. Images were selected from animals with matched plasma tracer and glucose concentrations. 5-HIAA and 5-HT content of the **(F)** dorsal and **(G)** caudal subdivisions of the DRN showed that the 5-HIAA:5-HT ratio was higher in CRFR2-null mice under basal conditions in the dorsal DRN (DRD) and was lowered by ARS only in CRFR2-null mice in both DRD and caudal DRN (DRC). Data expressed as mean ± SEM. *N* = 8 for LCMRglu, ANOVA critical *F*_(1,28)_ value = 4.196 for *P* ≤ 0.05, 7.636 for *P* ≤ 0.01, 13.500 for *P* ≤ 0.001. *N* = 6 or 7 for 5-HIAA and 5-HT content, ANOVA critical *F*_(1,22)_ values = 4.301 for *P* ≤ 0.05, 7.945 for *P* ≤ 0.01, 14.380 for *P* ≤ 0.001. *P < 0.05, **P < 0.01 in post-hoc analysis.

CRFR2 in the raphe nuclei modulate 5-HT activity, with consequences for stress-related behaviours [32-38]. Therefore serotonergic functions were examined in CRFR2-null and control mice. Neuronal metabolic activity, as measured by LCMRglu, was lower in both the DRN (*t* = 2.626, *P* = 0.048, *n* = 8 for 8-OH-DPAT experiment, *t* = 2.804, *P* = 0.036, *n* = 8 for DOI experiment) and median raphe nucleus (MRN) (*t* = 2.472, *P* = 0.049, *n* = 8 for 8-OH-DPAT experiment, *t* = 2.785, *P* = 0.038, *n* = 8 for DOI experiment) of CRFR2-null mice as compared with controls under basal conditions (Figure 4).

Following challenge with the 5-HT_1A_R-specific agonist 8-OH-DPAT, a main effect of treatment (ANOVA: *F*_(1,28)_ = 4.558, *P* = 0.044), and an interaction between genotype and treatment was observed in DRN (ANOVA: *F*_(1,28)_ = 5.953, *P* = 0.021) (Figure 4). Post-hoc analysis revealed that controls responded with decreased LCMRglu in both the DRN (*t* = 3.235, *P* = 0.0124, *n* = 8) and the MRN (*t* = 2.520, *P* = 0.047, *n* = 8) as expected, whereas the raphe nuclei of CRFR2-null mice were unresponsive to 5-HT_1A_R agonist. Following 5-HT_2_R-specific agonist DOI challenge, only a main effect of genotype was seen in both the DRN (ANOVA: *F*_(1,28)_ = 5.224, *P* = 0.030) and the MRN (ANOVA: *F*_(1,28)_ = 5.333, *P* = 0.029). The pattern of responses was, however, the same as for 8-OH-DPAT.

Studies to date have largely concentrated on the role of the DRN in respect to behaviour and anxiety. Therefore, we measured 5-HT and 5-HIAA within subregions of the DRN (Figure 4). Within the caudal DRN, there was a main effect of genotype on 5-HIAA (ANOVA: *F*_(1,22)_ = 7.094, *P* = 0.014) and a genotype X ARS interaction on 5-HIAA:5-HT ratio (ANOVA: *F*_(1,22)_ = 6.153, *P* = 0.021). Post-hoc analysis revealed an increase in 5-HIAA (*t* = 3.472, *P* = 0.002, *n* = 7) and 5-HIAA:5-HT ratio (*t* = 3.242, *P* = 0.004, *n* = 7) in CRFR2-mice under basal conditions, indicating increased serotonin turnover here. 24 hours following ARS, both the caudal (*t* = 2.759, *P* = 0.011, *n* = 6 or 7) and the dorsal (*t* = 4.087, *P* = 0.0005, *n* = 6 or 7) DRN showed decreases in 5-HIAA:5-HT ratio in CRFR2-null mice, with an associated decrease in 5-HIAA in the caudal DRN of CRFR2-null mice (*t* = 2.554, *P* = 0.018, *n* = 6 or 7), whereas controls showed no effect of ARS on these parameters.

### 5-HT responses to stress and 5-HTR agonists are altered in efferent brain regions of CRFR2-null mice

Following challenge with the 5-HT_1A_R-specific agonist 8-OH-DPAT, there was a main effect of treatment throughout the forebrain (ANOVA: *F*_(1,28)_ = 4.196 for *P* = 0.05) (Table 1) with a genotype × 8-OH-DPAT interaction observed in some extrapyramidal and limbic structures. Post-hoc analysis revealed that while controls had decreased LCMRglu in response to 8-OH-DPAT in extrapyramidal regions as expected, CRFR2-null mice showed no response. These areas receive projections from the DRN but lack their own 5-HT_1A_R, indicating that this reflects attenuated DRN response to 5-HT_1A_R agonist.

**Table 1:** **LCMRglu in efferent brain regions of control and CRFR2-null mice in response to 5-HT**_**1A**_**R or 5-HT**_**2**_**R agonist**

	**Control**			**CRFR2-null**			**Control**			**CRFR2-null**		
	**Saline**	**8-OH-DPAT**	**%**	**Saline**	**8-OH-DPAT**	**%**	**Saline**	**DOI**	**%**	**Saline**	**DOI**	**%**
**Neocortex**												
Frontal	46 ± 3	33 ± 3*	**−28**	47 ± 3	42 ± 3*	**−21**^a^	47 ± 3	38 ± 2*	**−19**	45 ± 2	30 ± 2*	**−33 **^a, b^
Anterior cingulate	48 ± 3	35 ± 2*	**−27**	49 ± 2	39 ± 4*	**−35**^a^	50 ± 3	40 ± 2*	**−15**	51 ± 3	34 ± 2*	**−33**^a^
Prefrontal	47 ± 3	35 ± 3*	**−26**	47 ± 3	42 ± 2	**−6**^a^	50 ± 3	39 ± 3*	**−22**	50 ± 3	30 ± 2*	**−40**^a^
Somatosensory	55 ± 2	33 ± 4*	**−40**	51 ± 4	45 ± 3*	**−22**^a^	56 ± 2	53 ± 3	**−5**	55 ± 4	44 ± 3*	**−20**^a^
Parietal	53 ± 2	35 ± 2*	**−34**	50 ± 4	43 ± 3	**−21**^a^	55 ± 2	45 ± 1*	**−18**	54 ± 3	37 ± 2*	**−31**^a, b^
**Hippocampus**												
Subiculum	40 ± 3	26 ± 3*	**−35**	45 ± 3	23 ± 3*	**−49**^a^	42 ± 2	43 ± 3	**2**	43 ± 3	33 ± 3*	**−23**
Dentate gyrus	26 ± 3	16 ± 3*	**−38**	30 ± 2	15 ± 3*	**−50**^a^	28 ± 3	18 ± 2*	**−36**	27 ± 3	16 ± 2*	**−14**^a^
*CA1*	37 ± 3	22 ± 4*	**−41**	38 ± 3	16 ± 2*	**−58**^a^	36 ± 3	26 ± 3*	**−28**	37 ± 2	26 ± 2*	**−30**^a^
*CA2*	35 ± 3	20 ± 3*	**−43**	36 ± 3	19 ± 2*	**−47**^a^	35 ± 3	34 ± 3	**−3**	36 ± 3	29 ± 2*	**−19**
*CA3*	38 ± 2	20 ± 2*	**−47**	41 ± 2	17 ± 2*	**−58**^a^	36 ± 2	28 ± 3	**−22**	36 ± 2	22 ± 3*	**−39**^a^
**Extrapyramidal areas**												
Medial striatum	43 ± 3	33 ± 2*	**−23**	42 ± 3	39 ± 3	**−7**^a^	45 ± 3	34 ± 2*	**−24**	46 ± 3	29 ± 2*	**−37**^a^
Lateral striatum	46 ± 3	29 ± 3*	**−37**	44 ± 3	41 ± 3	**−7**^c^	46 ± 4	30 ± 3*	**−35**	48 ± 4	31 ± 3*	**−35**^a^
Globus pallidus	33 ± 2	20 ± 2	**−39**	32 ± 2	33 ± 2	**−3**^a, b, c^	32 ± 2	30 ± 2	**−6**	35 ± 2	23 ± 3*	**−34**^a, c^
Substantia nigra	30 ± 3	20 ± 1*	**−27**	27 ± 3	25 ± 4	**−7**	28 ± 4	30 ± 2	**7**	28 ± 2	18 ± 4	**−36**
**Limbic areas**												
Medial septal nucleus	41 ± 3	30 ± 2*	**−27**	39 ± 3	22 ± 2*	**−44**^a^	45 ± 2	31 ± 2*	**−31**	48 ± 3	30 ± 2*	**−38**^a^
Lateral septal nucleus	38 ± 3	27 ± 3*	**−29**	40 ± 2	18 ± 2*	**−55**^a, c^	37 ± 3	28 ± 3	**−24**	40 ± 4	27 ± 3*	**−33**^a^
Bed nucleus of the stria terminalis	27 ± 4	18 ± 2*	**−33**	31 ± 2	16 ± 3*	**−48**^a^	30 ± 4	20 ± 2	**−33**	30 ± 2	18 ± 3*	**−40**^a^
Basolateral amygdala	36 ± 3	26 ± 3*	**−28**	36 ± 2	15 ± 2*	**−58**^a, b, c^	38 ± 2	27 ± 4	**−29**	38 ± 2	24 ± 4*	**−37**^a^
Central amygdala	26 ± 2	20 ± 2*	**−23**	25 ± 3	15 ± 2*	**−40**^a^	25 ± 2	17 ± 2	**−32**	24 ± 3	15 ± 2*	**−38**^a^

Local cerebral glucose utilization (LCMRglu), shown as mean ± SEM and percentage change in LCMRglu in 8-OH-DPAT or DOI compared with saline-treated mice. *n* = 8. ^a^Main effect of drug; ^b^main effect of genotype, ^c^genotype X drug interaction in 2-way ANOVA, where critical *F*_(1,28)_ value was 4.20 for *P* ≤ 0.05. *P < 0.05 vs saline in post-hoc analysis.

In limbic areas, both genotypes decreased LCMRglu significantly (Table 1), but the genotype × 8-OH-DPAT interaction in lateral septum (ANOVA: *F*_(1,28)_ = 4.654, *P* = 0.040) and basolateral amygdala (BLA) (ANOVA: *F*_(1,28)_ = 4.654, *P* = 0.040) revealed that the CRFR2-null mice had a greater response to 5-HT_1A_R agonist in these areas. Following DOI challenge, there was again a main effect of treatment throughout the forebrain (ANOVA: *F*_(1,28)_ = 4.196 for *P* = 0.05) (Table 1). Post-hoc analysis revealed that many brain regions showed a significant response to DOI in CRFR2-null mice but not controls (Table 1), suggesting greater postsynaptic 5-HT_2_R responsiveness throughout the forebrain in CRFR2-null mice.

**Figure 5:**
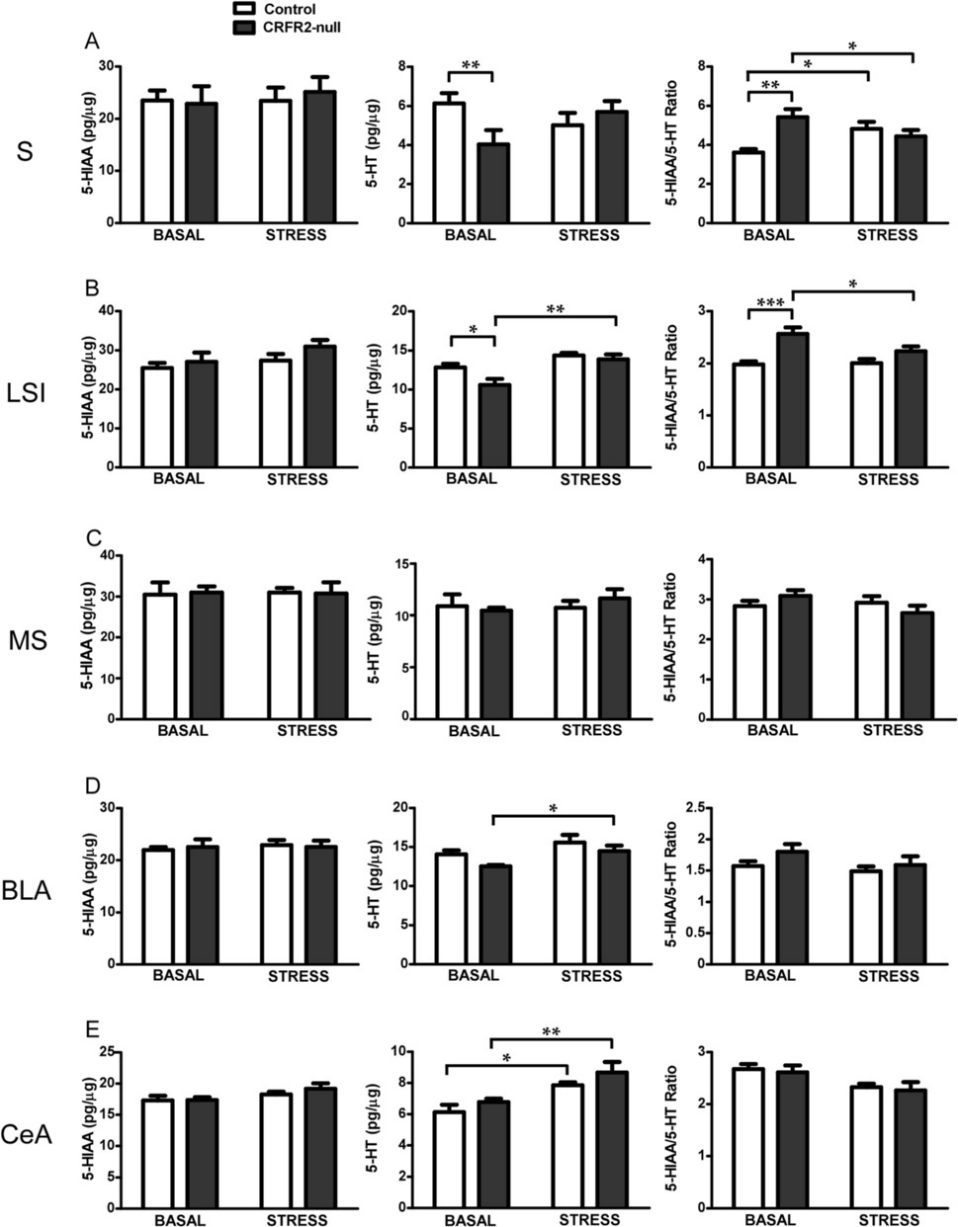
**5-HT responses to stress are altered in efferent brain regions of CRFR2-null mice.** 5-HIAA and 5-HT levels as well as 5-HIAA/5-HT ratios are shown in the **(A)** subiculum (S), **(B)** intermediate part of the lateral septum (LSI), **(C)** medial septum (MS), **(D)** basolateral amygdala (BLA) and **(E)** central amygdala (CeA). CRFR2-null mice showed differences in basal levels of 5-HT and or 5-HIAA:5-HT ratio in the S and LSI, and a differential response to stress in the LSI and BLA. Data expressed as mean ± SEM. *N* = 8 for LCMRglu, ANOVA critical *F*_(1,28)_ value = 4.196 for *P* ≤ 0.05, 7.636 for *P* ≤ 0.01, 13.500 for *P* ≤ 0.001. *N* = 6 or 7 for 5-HIAA and 5-HT content, ANOVA critical *F*_(1,22)_ values = 4.301 for *P* ≤ 0.05, 7.945 for *P* ≤ 0.01, 14.380 for *P* ≤ 0.001. * *P* < 0.05, ** *P* < 0.01, *** *P* < 0.001 in post-hoc analysis.

We then analyzed 5-HT and 5-HIAA content in the components of an anxiety-related amygdala-subiculum-septal circuit (Figure 5). There was a main effect of ARS on 5-HT content in the intermediate part of the lateral septum (LSI) (ANOVA: *F*_(1,22)_ = 15.41, *P* = 0.0008) and of genotype on the 5-HIAA:5-HT ratio (ANOVA: *F*_(1,22)_ = 19.460, *P* = 0.0002). There was also a genotype × ARS interaction in subiculum on both 5-HT (ANOVA: *F*_(1,22)_ = 5.196, *P* = 0.033) and 5-HIAA:5-HT ratio (ANOVA: *F*_(1,22)_ = 10.87, *P* = 0.004), and a main effect of genotype on 5-HIAA:5-HT (ANOVA: *F*_(1,22)_ = 4.585, *P* = 0.045).

Post-hoc analysis revealed that under basal conditions the 5-HIAA:5-HT ratio was increased in CRFR2-null mice (subiculum; *t* = 3.846, *P* = 0.001, *n* = 6: LSI; *t* = 4.657, *P* < 0.0001, *n* = 7). However, in contrast with the DRN, this was due to lower 5-HT (subiculum; *t* = 2.474, *P* = 0.022, *n* = 6-7: LSI; *t* = 2.759, *P* < 0.012, *n* = 7) with unchanged 5-HIAA. In response to ARS, 24 h later there was an increase in 5-HT in the LSI of CRFR2-null mice (*t* = 3.878, *P* = 0.0009, *n* = 6 or 7) and a decrease in the 5-HIAA:5-HT ratio (*t* = 2.516, *P* = 0.020, *n* = 6 or 7). The genotype x ARS interaction in the subiculum was such that 5-HIAA:5-HT was increased by ARS in controls (*t* = 2.569, *P* = 0.018, *n* = 6 or 7), but decreased in CRFR2-null mice (*t* = 2.094, *P* = 0.049, *n* = 6 or 7). In the CeA, there was a main effect of ARS (ANOVA: *F*_(1,22)_ = 17.71, *P* = 0.004) to increase 5-HT content in both genotypes (*t* = 2.838, *P* = 0.010, *n* = 6 or 7 for controls; *t* = 3.113, *P* = 0.005 for CRFR2-null mice, *n* = 6 or 7). 5-HT levels also increased in the BLA (*t* = 2.168, *P* = 0.041, *n* = 6 or 7) of CRFR2-null mice in response to ARS (Figure 5).

### Serotonergic and corticosteroid receptor gene expression are altered in response to stress in CRFR2-null mice

To investigate which factors potentially involved in the processes of adaptation to acute stress might be differentially regulated in CRFR2-null mice as compared with controls, SERT protein levels (ligand binding) and mRNA levels of serotonergic genes and corticosteroid receptors (ISH) were quantified in apposite brain nuclei following ARS or end of CVMS. A time of 12 h post-stress was chosen as appropriate, as altered expression of these factors at this time has previously been observed by many investigators. Full results are in Additional file 1; only key significant differences are presented here.

**Figure 6:**
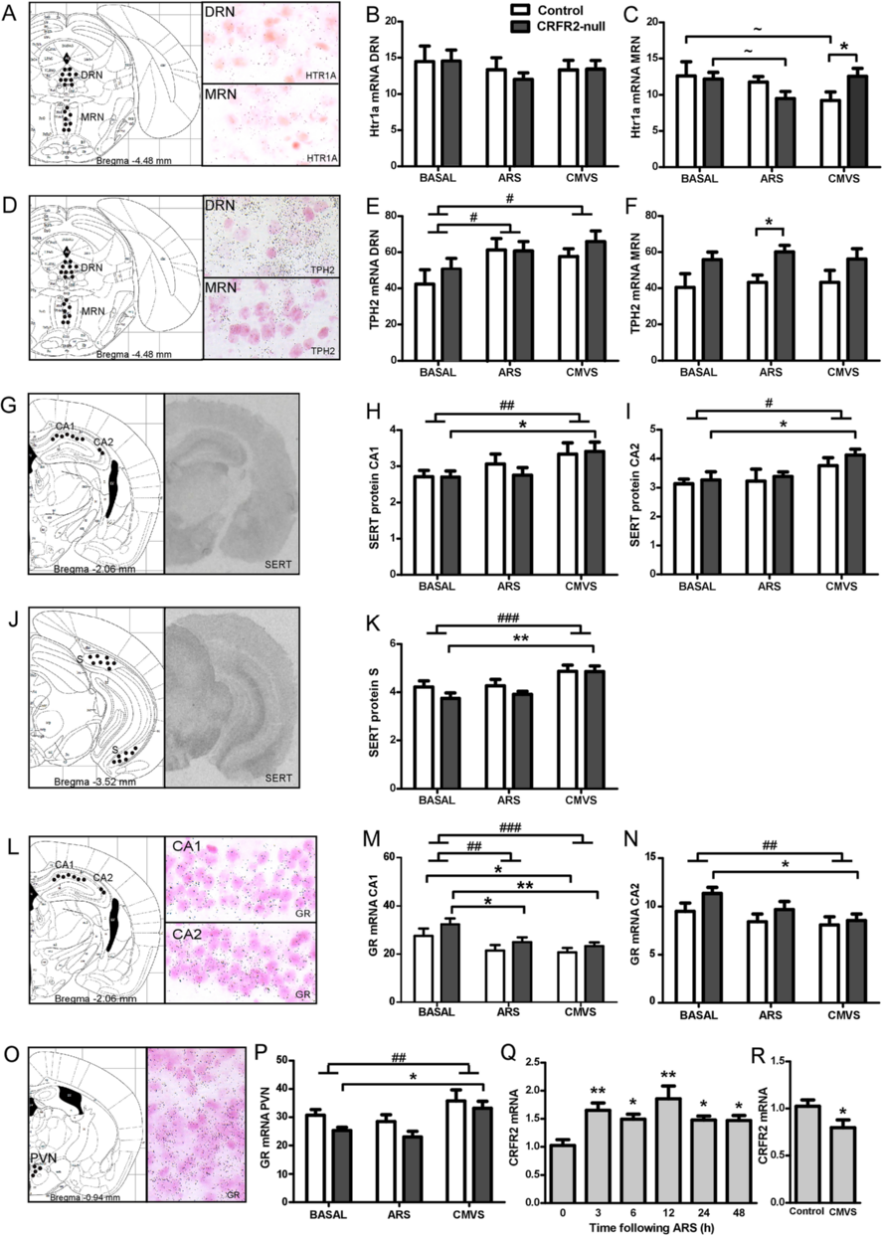
**Serotonergic and corticosteroid receptor expression are regulated differentially in response to stress in CRFR2-null mice. (A)** mRNA expression of 5-HT_1A_R in the **(B)** dorsal raphe nucleus (DRN) and **(C)** median raphe nucleus (MRN); **(D)** tryptophan hydroxylase 2 (TPH2) mRNA in **(E)** DRN and **(F)** MRN; **(G)** serotonin transporter (SERT) protein expression in **(H)** hippocampal CA1, **(I)** CA2 and **(J,K)** subiculum (S); **(L)** glucocorticoid receptor (GR) mRNA expression in **(M)** CA1, **(N)** CA2 and **(O,P)** the paraventricular nucleus (PVN). Data presented as mean ± SEM for mRNA levels or densitometry signal (SERT). *N* = 6 to 8. ANOVA critical *F*_(2,36)_ value = 3.259 for *P* ≤ 0.05, 5.248 for *P* ≤ 0.01, 8.420 for *P* ≤ 0.001. ^#^*P* < 0.05, ^##^*P* < 0.01, ^###^*P* < 0.001 for effect of stress across genotypes. *P < 0.05, **P < 0.01, ***P < 0.001 ~*P* <0.10 in post-hoc analysis. ARS increased CRFR2 mRNA expression in whole brain of mice at all times points up to 48 h post-stress **(Q)**, whereas CVMS decreased CRFR2 expression 7 days after the end of the CVMS protocol **(R)***.* Data presented as mean ± SEM. *N* = 8. ANOVA critical *F*_(5,42)_ value = 2.438 for *P* ≤ 0.05, 3.488 for *P* ≤ 0.01. *P < 0.05, ** *P* < 0.01 as compared with 0 hours in post-hoc analysis or with control group.

In agreement with responses to 8-OH-DPAT, 5-HT_1A_R mRNA expression did not differ with genotype in the hippocampus or amygdala (Additional file 1). No effect of genotype or stress was seen in the DRN (Figure 6), but a genotype x stress interaction (ANOVA: *F*_(2,36)_ = 3.328, *P* = 0.048) whereby decreased expression in control compared with CRFR2-null mice (*t* = 2.181, *P* = 0.036, *n* = 7) was seen in the MRN following CVMS, and there were trends for ARS to reduce 5-HT_1A_R expression in CRFR2-null mice (*t* = 1.702, *P* = 0.098, *n* = 6 or 7) but not controls, and for CVMS to reduce 5-HT_1A_R expression in controls alone (*t* = 2.020, *P* = 0.052, *n* = 6 or 7). There was no appreciable effect of genotype on 5-HT_2C_R mRNA expression (Additional file 1).

There was a main genotype effect on TPH2 mRNA expression in MRN (ANOVA: *F*_(2,36)_ = 5.311, *P* = 0.027), with increased levels in CRFR2-null mice. Post-hoc analysis detected this as significant only between ARS groups (*t* = 2.080, *P* = 0.045, *n* = 6 or 7) (Figure 6). There was a main effect of stress on TPH2 in DRN (ANOVA: *F*_(2,36)_ = 3.684, *P* = 0.036) across genotypes.

In the hippocampus, there was a main effect of stress on SERT protein expression (CA1 ANOVA: *F*_(2,36)_ = 4.106, *P* = 0.027; CA2 ANOVA: *F*_(2,36)_ = 4.387, *P* = 0.020; subiculum ANOVA: *F*_(2,36)_ = 8.474, *P* = 0.001), owing to increased expression after the end of CVMS, only reaching statistical significance in CRFR2-null mice (CA1 *t* = 2.151, *P* = 0.038, *n* = 7 or 8; CA2 *t* = 2.139, *P* = 0.026, *n* = 7 or 8; subiculum *t* = 3.490, P = 0.0013, *n* = 7 or 8) and not in controls (Figure 6). There were no effects of genotype or stress on SERT expression in the amygdala (Additional file 1).

Expression of GR mRNA showed differential effects between brain regions and genotypes (Figure 6). In the dorsal hippocampus, there was a trend towards higher GR expression in CRFR2-null mice (CA1 ANOVA: *F*_(2,36)_ = 3.976, *P* = 0.054; CA2 ANOVA: *F*_(2,36)_ = 4.008, *P* = 0.067). There was a main effect of stress (ANOVA: *F*_(2,36)_ = 7.312, P = 0.002) with both ARS and CVMS reducing expression in CA1 of CRFR2-null mice (ARS *t* = 2.420, *P* = 0.021, *n* = 7 or 8; CVMS *t* = 2.962, *P* = 0.005, *n* = 7 or 8), but only CVMS had a significant effect in controls (*t* = 2.962, *P* = 0.043, *n* = 6 or 7). In CA2, only CVMS had an effect to reduce GR expression, and this was only significant in CRFR2-null mice (*t* = 2.725, *P* = 0.010, *n* = 7 or 8). In the paraventricular nucleus (PVN) there was a main effect of genotype (ANOVA: *F*_(2,36)_ = 6.788, *P* = 0.003) with CRFR2-null mice having lower GR mRNA expression, although this was not significant within treatment groups in post-hoc analysis. There was a main effect of stress (ANOVA: *F*_(2,36)_ = 4.974, *P* = 0.032); post-hoc analysis showed increased GR following CVMS, but only reaching significance in CRFR2-null mice (*t* = 2.341, *P* = 0.025, *n* = 7 or 8). Expression of MR mRNA was not regulated by stress or genotype in any brain region examined (Additional file 1). Finally, CRFR2 mRNA levels, as quantified by qPCR, were increased over a time period of 3 to 48 hours post-ARS (ANOVA: *F*_(5,42)_ = 3.750, *P* = 0.007) but were decreased following CVMS (*t* = 2.164, *P* = 0.047, *n* = 8) (Figure 6).

## Discussion

This study extends the evidence regarding the importance of CRFR2 in mediating the processes towards successful behavioural recovery in the period following stress, and moreover demonstrates that CRFR2 is engaged in the control of serotonergic function during the same time frame. It further characterizes the stress-sensitive phenotype of CRFR2-null mice [50,51,62] and reveals fundamental disturbances within components of their serotonergic system.

In contrast with original reports of increased basal levels of anxiety [50,51], in our hands, similar to the findings of Coste *et al.*[62], CRFR2-null mice do not show increased anxiety-like behaviour compared to controls until 24 h after exposure to a prior acute stressor. This discrepancy could be due to differing phenotypes of the three independently generated strains of CRFR2-null mice, or factors such as age or husbandry. However, the mice in this study are the same strain as reported with increased anxiety by Bale *et al.*[50] and an anxious phenotype was described for both group- [50] and singly [51] housed CRFR2-null mice from 9 [40] to 24 [50] weeks of age, but not at 16 weeks [62], meaning that these factors are unlikely to explain the inconsistency. This study indicates the need for a prior stress for increased anxiogenesis in CRFR2-null mice, so an alternative explanation is that mice in previous studies might have been inadvertently previously stressed, for example, by a prior behavioural test. This time course of the behavioural effects of ARS led us to conclude that CRFR2 has a key role in the processes leading to behavioural recovery in the hours following exposure to a stressor.

While CRFR2-null mice in our study appear to be in a maladaptive state at 24 hours following an acute stress, CRFR2-null mice exposed to CVMS are not more anxious than controls. It could be interpreted from this that CRFR2-null mice have the ability to cope successfully with this more chronic stress, but it is more likely that both CRFR2-null mice and controls are affected adversely by CVMS, while CRFR2-null mice show an exaggerated response to a single acute stressor. Such stressors might release CRF sufficient to recruit CRFR2 [20], which mediate successful stress coping in normal mice [63]. Alternatively, increased CRFR1 signalling in response to stress might occur; increased CRF expression in the amygdala and PVN of CRFR2-null mice has been reported [50]. However, the time frame of delayed anxiogenesis in CRFR2-null mice does not correlate with the expected rapid release of CRF in response to acute stress and its subsequent negative feedback. The time interval required for CRFR2-null mice to acquire this anxiety trait suggests that the processes are indirect, and the serotonergic system is an obvious candidate.

Exogenous CRF administered to the DRN inhibits firing of 5-HT neurons via CRFR1 [15,18], while Ucns or higher levels of CRF increase firing via CRFR2 [32-36]. The raphe nuclei receive inputs from both CRF and Ucn1 neurons [14,15,29], which may therefore regulate serotonergic raphe function physiologically. In support of this hypothesis, CRFR2-null mice show altered 5-HT/5-HIAA content in the DRN, LSI, subiculum, CeA and BLA 24 h after ARS, whereas control mice showed a clear change only in 5-HT content of the CeA. Recent studies of mice with genetically altered Ucn levels have shown that 5-HT function is dysregulated in these models [39-41] and that CRFR2-null mice show greater sensitivity to elevation of 5-HT levels by pharmacological means, an observation suggested to be linked to their stress-sensitive phenotype [64]. Notably, mice deficient in all three Ucns do indeed show a similar phenotype [41] to our observations in CRFR2-null mice, with increased anxiety-like behaviour and dysregulated activity within 5-HT circuits 24 h following ARS, again evidencing the importance of CRFR2 here.

Interestingly, CRFR2-null mice show decreased basal neuronal metabolic activity in the raphe nuclei. This is typically interpreted as evidence of decreased 5-HT firing activity levels, as while both 5-HT and GABAergic neurons are important functionally here, GABAergic neurons are present at only 10% of the number of 5-HT neurons [65]. This is an unusual finding under basal conditions in our experience and could be due to increased raphe 5-HT_1A_R inhibitory autoreceptor activity, altered 5-HT_1A_R modulation of raphe GABAergic interneurons that express both CRFR2 and 5-HT_1A_R, or by inhibition from forebrain postsynaptic receptors including 5-HT_1A_R and 5-HT_2_R [66-69]. Increased sensitivity of structures throughout the forebrain to 5-HT_2_R agonists and to 5-HT_1A_R in some limbic structures in CRFR2-null mice suggests that postsynaptic receptor responsiveness is increased, and thus the latter mechanism may be significant. A shift towards unopposed CRFR1 activity in the raphe nuclei of CRFR2-null mice could also be a significant factor in mediating these effects or directly inhibiting 5-HT neuronal activity. Uncontrollable stress, which activates DRN serotonergic neurons [38], is associated with a functional desensitization of 5-HT_1A_R [70]. We observed no significant differences in 5-HTR expression in the DRN of CRFR2-null mice and so it is likely that these effects are also mediated by decreased internalization and desensitization of receptors [71], providing a mechanism for potentially very dynamic responses to stress. Detailed electrophysiological studies would be required to resolve the mechanism further.

In contrast with forebrain structures, the lack of LCMRglu response in the raphe nuclei to 5-HTR agonists in CRFR2-null mice suggests tonic inhibition of neuronal activity here may be close to maximal under basal conditions. The response to 5-HT_1A_R agonist in extrapyramidal brain areas receiving projections from the DRN [72,73] but lacking their own 5-HT_1A_R [74,75] was also attenuated. Thus CRFR2 appear to be required for maintaining normal basal neuronal activity in the raphe nuclei and, in particular, for the balance of 5-HT_1A_R function here.

CRFR2 are present in both the DRN and the MRN [11,13]. However, studies of stress biology have largely concentrated on the DRN, and so to relate this altered raphe function to the CRFR2-null behavioural phenotype, we examined 5-HT responses to stress in the DRN and associated anxiety-related nuclei. Concentrations of 5-HIAA and the 5-HIAA:5-HT ratio were elevated in CRFR2-null mice under baseline conditions within the caudal subregion of the DRN (DRC), and these effects approached significance in the adjacent dorsal subregion (DRD). The DRD and DRC mediate CRF receptor responses and are considered to be anxiety-related subregions of the DRN based on anatomical and functional criteria [76,77]. For example, they are activated by anxiogenic drugs [78], CRF-related peptides *in vivo*[33,79] and *in vitro*[80], inescapable shock [70], noise stress [80], social defeat [81], the avoidance task on the elevated T-maze [82], acoustic startle [83] and anxiety due to prior experience of intimate partner violence [84]. In support of the specificity of these anxiety-related effects on DRD/DRC serotonergic systems, in none of these studies were serotonergic neurons in the adjacent ventrolateral part of the DRN activated. Elevation of 5-HIAA and 5-HIAA:5-HT ratios in the DRC could be due to an organizational difference in 5-HT systems as a consequence of CRFR2-null phenotype, or to differential activity that develops later in life. In either case, DRC neurons appear to have altered baseline activity in adult CRFR2 null mice, which may reflect a vulnerability to increased anxiety states.

Despite lower 5-HT content in efferent stress-related nuclei under unstressed conditions in CRFR2-null mice, stress had a greater effect on their 5-HT content at 24 h, in keeping with their stress-sensitive phenotype. This was particularly evident in LSI, which receives significant 5-HT projections from the caudal DRN [85,86], in the subiculum and, to a lesser extent, in the CeA. The subiculum is a key structure in inhibiting the hypothalamic-pituitary-adrenal axis (HPAA) during termination of the stress response [87] and so altered function here might relate to the higher responsiveness of the HPAA in CRFR2-null mice following acute stress [50,62].

Not all anxiety-related nuclei examined showed such changes. We found no differences in the LCMRglu of the bed nucleus of the stria terminalis (BNST) between control and CRFR2-null mice at either baseline or in response to 8-OH-DPAT or DOI challenge. This was unexpected, given the pivotal role of the BNST in the control of anxiety states [88]. However, the serotonergic dysregulation in CRFR2-null mice may be downstream of the BNST which projects strongly to the DRD/DRC region [89], where CRFR2 are abundant [12,13]. Overexpression of CRF in the BNST induces a decrease in CRFR2 binding selectively in the DRD/DRC [90] and so it may be that the observed effects in CRFR2-null mice are primarily mediated here.

5-HT firing activity is generally increased by stress [20] and negative feedback to the DRN ultimately restores balance [66-69,71], as evidenced by essentially unchanged 5-HT and 5-HIAA levels in control mice at 24 h following ARS. It has been previously reported that CRFR2-null mice show greater sensitivity to 5-HT modulation of stress-induced behaviours [64]. The pattern of increased responses in CRFR2-null mice to both 5-HT_1A_R and 5-HT_2_R receptor agonists in areas expressing postsynaptic receptors is in keeping with this finding. The LSI and BLA are key components of the limbic stress circuitry that were more responsive to 5-HT_1A_R agonist in CRFR2-null mice. This might therefore relate to their stress-sensitive phenotype and indicate a role for these structures in stress recovery.

The MRN has been implicated in mediating a delayed coping response following fear behaviour induced by CRF in the DRN [35,91]. A delayed increase in 5-HT in the mPFC mediated by CRFR2 in MRN is associated with cessation of intra-DRN CRF-induced freezing behaviour [91] and is therefore proposed to mediate stress resilience effects [92]. In CRFR2-null mice, the CRFR2-mediated surges in 5-HT neuronal firing from DRN and MRN cannot occur, and unopposed CRFR1-mediated inhibition in DRN might further contribute to this [15,18]. The normal 5-HT response in mPFC occurs one to two hours after intra-DRN CRFR2 receptor activation [35,91], and we infer that the maladaptive state in CRFR2-mice develops after the peak of this CRFR2-induced increase in mPFC 5-HT and by 24 h after the stress. Thus, we propose that this delayed activity in efferent 5-HT neurons, which is critical for successful adaptation to acute stress, is disrupted in CRFR2-null mice. The consequences for CRFR2-null mice beyond 24 h are unknown, but unlike controls, 5-HT levels at this time point are increased from basal levels in several limbic nuclei, indicating that homeostasis has not been restored It is feasible that a lack of negative feedback within the 5-HT system due to the failure of CRFR2-mediated 5-HT activity might contribute to this.

**Figure 7:**
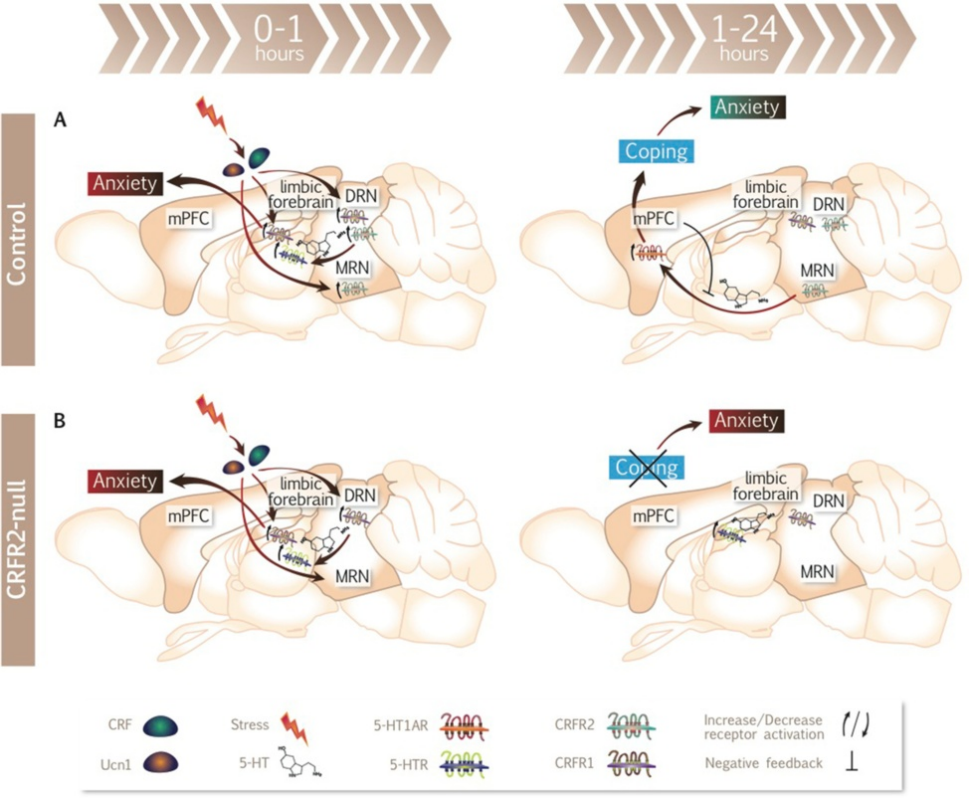
**Proposed model for development of prolonged anxiety following acute stress in CRFR2-null mice.** Following acute stress in control mice **(A)** CRF acting at CRFR1 in the limbic forebrain produces immediate anxiety. High levels of CRF and potentially Ucn1 activate CRFR1 and more abundant CRFR2 in the DRN with a net effect to promote early firing of efferent 5-HT neurons to limbic nuclei. Activation of CRFR2 in the MRN promotes delayed 5-HT release in the mPFC at 1 to 2 h, which acts at 5-HT1AR to mediate successful coping and anxiolysis by 24 h. Negative feedback in the 5-HT system restores homeostasis by 24 h. **(B)** In CRFR2-null mice, the CRFR2-mediated increase in 5-HT firing cannot occur and unopposed CRFR1 activity might inhibit 5-HT neuronal firing in limbic nuclei even further. Absence of negative feedback within the 5-HT system contributes to the increased 5-HT levels observed in limbic areas at 24 h. The temporal dynamics of the 5-HT system following acute stress are dysregulated and homeostasis has not been restored. Crucially, the delayed 5-HT activity in mPFC is disrupted and successful coping has not occurred, resulting in prolonged anxiety.

Therefore, we propose that a rapid and highly regulated increase in CRFR2 signalling in response to acute stress, the resultant increase in efferent 5-HT activity and subsequent negative feedback to restore homeostasis are important for a normal and successful coping response. The delayed increase in 5-HT in mPMC is of key importance. Without this orchestrated response, CRFR2-null mice do not respond to stress appropriately, and there is prolonged anxiety that might account for their well-recognized anxiety phenotype. This proposed model is presented in Figure 7. There is significant evidence for a role for the MRN in stress recovery [91,92], and this model is consistent with our observation that LCMRglu is lower under basal conditions in the MRN of CRFR2-null mice while the robust elevation of TPH2 mRNA in CRFR2-null MRN might be a compensatory response to a lack of CRFR2 activation here. More detailed analysis of the dynamics of CRF and 5-HT processes in this time frame and beyond in appropriate subregions of the raphe nuclei, and consideration of the roles of other mediators of the stress response in CRFR2-null mice, are required to substantiate this further.

Owing to the number of mediators involved in stress responses and the complex interactions among them, other factors in addition to the serotonergic system are likely to be modified in CRFR2-null mice in the hours following stress exposure, which might have implications for the longer term. Indeed Ucn1 expression in the Edinger-Westphal nucleus and CRF in CeA (but not the PVN) are increased in CRFR2 mice [50], which may be a developmental compensatory change that is also responsible at least in part for their phenotype. Expression of CRFR1 is, however, unaltered. We found that changes in serotonergic and corticosteroid receptor gene expression in response to stress were generally greater in CRFR2-null mice, again in keeping with their stress-sensitive behavioural phenotype. CRFR2-null mice have normal basal HPAA activity, but higher responsiveness following acute stress [50,62]. Hence, changes such as the observed greater stress-induced increases in hippocampal SERT levels in CRFR2-null mice may be mediated by glucocorticoids [93,94], adding potentially further complexity to the relationship between CRFR2 and 5-HT function. Stress also downregulated hippocampal GR mRNA to a greater degree in CRFR2-null mice, potentially reflecting this expected HPAA hyperactivation. CRFR2-null mice also had lower basal GR expression in the PVN, possibly reflecting chronically higher HPAA tone, and CVMS unexpectedly increased this. Discordant regulation of GR expression in the hippocampus and PVN has been reported previously [95,96], with upregulation of GR by stress suggested to maintain glucocorticoid signalling to limit HPAA responses during prolonged stress. 5-HT also regulates GR expression, and this may be mediated through TPH2 activity, in order to regulate HPAA activity [97]. Both TPH2 and 5-HT_1A_R mRNAs in MRN were differentially expressed in CRFR2-null mice. TPH2 mRNA levels in MRN were higher in CRFR2-null mice, and there may be altered afferent control of DRN activity from here [98], suggesting that the MRN should be more carefully considered in future studies of CRFR2 function.

Given this proposed role of CRFR2, we might expect expression to be regulated by stress exposure. We found expression to increase, reaching a maximum at 3 to 12 h post-ARS and subsequently declining, an effect similar to that seen for CRFR1 when acutely exposed to ligand [99], while chronic stress decreased CRFR2 mRNA expression in this and a previous study [100]. Others have observed lower CRFR2 expression in adult rats subjected to maternal deprivation or in genetically stress-sensitive rodent strains [101,102], suggesting that CRFR2 downregulation has the potential to be permanent in anxious or stress-sensitive animals. The interesting exceptions are where CRFR2 is increased by chronically elevated levels of CRF [103] or corticosterone [104], or in a model of maladaptive post-traumatic stress disorder-like behaviour [105]. We hypothesize that while increased CRFR2 activity is required for successful recovery from stress and subsequent downregulation is a normal adaptive response associated with healthy coping, that ongoing hyperactivity of CRFR2 might be associated with a maladaptive stress response. The role of CRFR2 in mediating learned helplessness in response to uncontrollable stress has implicated CRFR2 activity in the development of maladaptive behavioural responses [38,79]. However equally, CRFR2 upregulation might be an appropriate secondary adaptation to a chronic stress. This issue requires further investigation, to assess whether CRFR2 is a potential target in stress-related psychiatric disorders.

In this study, 5-HT function in the lateral septum and subiculum, sites linked with anxiety as well as the neural circuitry of reward and addiction [106-108], was particularly altered. Dysregulated serotonergic function has long been linked to stress-related psychopathologies [109,110] and direct effects of Ucns on CRFR2 in LSI have been observed in rodent models of these disorders; hence, CRFR2 may play an important role in these processes [111-113].

## Conclusions

While the role of CRFR2 in stress recovery was proposed some time ago [63,114], this study provides new information regarding the mechanisms by which this may be mediated and highlights the importance in the immediate post-stress period. This has implications for the pathophysiology of psychiatric conditions associated with acute stress exposure, such as post-traumatic stress disorder, reactive depression and relapse to substance abuse. As evidence continues to emerge that CRFR2 may mediate its effects on stress primarily through 5-HT, the potential for involvement in further mood disorders and ultimately for therapeutic targeting is clear.

## Abbreviations

5-HT: serotonin; 5-HTR: 5-HT receptor; 5-HIAA: 5-hydroxyindoleacetic acid; 8-OH-DPAT: 8-hydroxy-*N*,*N*-dipropyl-2-aminotetralin; ANOVA: analysis of variance; ARS: acute restraint stress; BLA: basolateral amygdala; BNST: bed nucleus of the stria terminalis; CeA: central amygdala; CVMS: chronic variable mild stress; CRF: corticotropin-releasing factor; CRFR1: corticotropin-releasing factor type 1 receptors; CRFR2: corticotropin-releasing factor type 2 receptors; DOI: 1-(2,5-dimethoxy-4-iodophenyl)-2-aminopropane; DRC: dorsal raphe nucleus, caudal part; DRD: dorsal raphe nucleus, dorsal part; DRN: dorsal raphe nucleus; GR: glucocorticoid receptor; HPAA: hypothalamic-pituitary-adrenal axis; ISH: *in-situ* hybridization histochemistry; LCMRglu: local cerebral glucose utilization; LDT: light/dark transfer test; LSI: intermediate part of the lateral septum; MR: mineralocorticoid receptor; MRN: median raphe nucleus; MS: medial septum; OF: open-field; PVN: paraventricular nucleus of the hypothalamus; qPCR: quantitative polymerase chain reaction; S: subiculum; SEM: standard error of the mean; SERT: serotonin transporter; TPH2: tryptophan hydroxylase 2; Ucn: urocortin.

## Competing interests

The authors declare that they have no competing interests.

## Authors’ contributions

PMJ acquired funding, designed the study, performed experiments, analyzed and interpreted data and wrote the manuscript. OI designed and performed experiments, analyzed and interpreted data and prepared figures. RNC, EDP, AN-C and YK performed experiments and analyzed data. PATK and HJO designed and performed experiments and analyzed and interpreted data. CAL acquired related funding, designed experiments and analyzed and interpreted data. JRS and AC acquired related funding and participated in study design and data interpretation. All authors provided comments and suggestions during manuscript preparation. All authors read and approved the final manuscript.

## Supplementary Material

Additional file 1 Serotonergic and corticosteroid gene expression in control and CRFR2-null mice in response to ARS or CVMS. Click here for file
