# Supplementary material for: Increased anxiety in corticotropin-releasing factor type 2 receptor-null mice requires recent acute stress exposure and is associated with dysregulated serotonergic activity in limbic brain areas
Source: Biol Mood Anxiety Disord. 2014 Jan 21;4:1. doi: 10.1186/2045-5380-4-1 (PMC4029322; doi:10.1186/2045-5380-4-1)
Supplement: Additional file 1 — Serotonergic and corticosteroid gene expression in control and CRFR2-null mice in response to ARS or CVMS. [file 2045-5380-4-1-S1.pdf]

Additonal File 1

Serotonergic and Corticosteroid gene expression in control and CRFR2-null mice in response to ARS or CMVS.

	Control			CRFR2-null		
	**Basal**	**ARS**	**CMVS**	**Basal**	**ARS**	**CMVS**
Htr1a						
Hippocampus						
CA1	11.85±1.29	9.26±1.23	9.76±1.13	11.94±1.89	10.07±0.76	9.75±0.67
CA2	6.57±0.81	6.94±1.33	6.01±0.86	7.05±0.65	7.11±0.76	6.75±0.58
CA3	5.70±0.53	5.31±0.60	5.47±0.72	5.75±0.55	4.51±0.17	5.18±0.46
CA4	5.68±0.74	5.77±0.42	4.96±0.79	5.47±0.65	4.97±0.53	5.05±0.48
DG ƒ	6.38±0.29	5.47±0.39	5.17±0.75	6.36±0.44	5.40±0.31	5.20±0.36
Amygdala						
CeA	4.32±0.51	5.03±0.58	4.83±0.64	4.21±0.26	5.14±0.47	5.18±0.49
MeA	4.24±0.74	5.21±0.30	5.26±0.28	5.19±0.48	4.73±0.55	4.85±0.34
BMA	4.32±0.50	5.24±0.67	4.57±0.36	4.35±0.45	4.56±0.62	4.23±0.17
BLA	4.34±0.57	4.57±0.38	4.50±0.54	3.77±0.37	5.51±0.76	4.4±0.39
Htr2c						
Hippocampus						
CA1	8.72±0.38	11.49±1.40	11.71±1.16	11.54±1.89	8.67±1.82	13.7±1.49
CA2	8.82±0.76	12.89±2.12	10.97±1.70	12.21±2.23	12.78±2.31	16.09±3.04
CA3	10.32±1.11	13.24±1.42	13.33±2.08	10.91±1.84	12.48±3.15	12.84±1.67
DG §	5.38±1.22	8.07±1.10	6.15±0.68	6.64±1.08	4.17±1.33	6.87±1.09
Amygdala						
CeA	21.33±3.83	24.79±1.91	23.21±3.18	21.93±3.70	21.03±3.30	28.84±4.22
MeA	30.22±3.40	38.69±5.16	37.57±5.72	29.27±4.87	31.77±1.57	39.19±6.01
BMA	27.06±2.46	23.99±3.03	26.66±2.59	22.87±3.36	23.98±2.14	26.26±2.86
BLA	19.78±3.18	20.94±2.89	24.73±3.24	21.33±4.38	14.08±1.93	19.6±2.52
SERT						
Amygdala						

CeA	0.06±0.01	0.06±0.01	0.07±0.01	0.06±0.01	0.06±0.01	0.06±0.01
MeA	0.07±0.01	0.08±0.01	0.07±0.00	0.07±0.00	0.07±0.01	0.07±0.01
BMA	0.12±0.01	0.13±0.01	0.13±0.00	0.13±0.01	0.12±0.01	0.12±0.01
BLA	0.14±0.01	0.14±0.01	0.15±0.00	0.14±0.01	0.14±0.01	0.14±0.01
GR						
Hippocampus						
CA3	10.99±1.27	11.49±1.15	9.12±0.93	12.26±0.93	12.20±1.21	10.26±1.05
CA4	10.12±1.14	9.97±1.09	8.84±1.22	12.35±1.57	12.00±0.68	9.48±0.86
DG	18.20±1.21	17.80±1.71	15.62±1.90	19.72±1.46	20.54±1.72	16.25±1.04
MR						
Hippocampus						
CA1	33.00±2.61	36.60±4.33	34.50±3.44	32.08±3.29	36.01±3.19	29.91±2.42
CA2	34.06±1.90	37.68±3.80	35.70±3.51	33.33±2.64	37.71±4.38	43.87±2.89
CA3	25.58±1.41	27.81±3.07	25.69±1.71	25.50±1.22	30.41±1.85	31.67±1.56
CA4	21.53±2.62	24.27±3.18	22.85±1.26	24.18±0.99	25.65±2.10	28.20±1.60
DG	28.20±2.17	30.92±2.99	29.07±1.99	30.49±2.20	31.41±3.02	30.97±2.13

Data are presented as mean ± SEM arbitrary units for hybridisation (Htr1a, Htr2c, GR, MR mRNAs) or densitometry signal (SERT). †Main effect of genotype, ƒMain effect of stress, §Main effect of genotype x stress in 2-way ANOVA. n=6-7.
SERT, 5-HT transporter; GR, glucocorticoid receptor; MR, mineralocorticoid receptor; DRN, dorsal raphé nucleus; MRN, median raphé nucleus; DG, dentate gyrus, LSI, lateral septum, intermediate part; MS, medial septum; CeA, central amygdala; MeA, medial amygdala; BMA, basomedial amygdala; BLA, basolateral amygdala; PVN, paraventricular nucleus of the hypothalamus.
